# Adenosine-5′-Phosphosulfate- and Sulfite Reductases Activities of Sulfate-Reducing Bacteria from Various Environments

**DOI:** 10.3390/biom10060921

**Published:** 2020-06-17

**Authors:** Ivan Kushkevych, Daryna Abdulina, Jozef Kováč, Dani Dordević, Monika Vítězová, Galyna Iutynska, Simon K.-M. R. Rittmann

**Affiliations:** 1 Department of Experimental Biology, Faculty of Science, Masaryk University, Kamenice 753/5, 62500 Brno, Czech Republic; jozef.kovac@mail.muni.cz (J.K.); vitezova@sci.muni.cz (M.V.); 2 Department of Molecular Biology and Pharmaceutical Biotechnology, University of Veterinary and Pharmaceutical Sciences Brno, 61242 Brno, Czech Republic; 3 Department of General and Soil Microbiology, D.K. Zabolotny Institute of Microbiology and Virology of the National Academy of Sciences of Ukraine, Acad. Zabolotnogo str. 154, 03143 Kyiv, Ukraine; abdulinadarina@gmail.com (D.A.); galyna.iutynska@gmail.com (G.I.); 4 Department of Plant Origin Foodstuffs Hygiene and Technology, Faculty of Veterinary Hygiene and Ecology, University of Veterinary and Pharmaceutical Sciences, 61242 Brno, Czech Republic; dani_dordevic@yahoo.com; 5 Archaea Physiology & Biotechnology Group, Department of Functional and Evolutionary Ecology, Universität Wien, Althanstraße 14, 1090 Vienna, Austria

**Keywords:** hydrogen sulfide, toxicity, sulfate-reducing bacteria, cell-free extracts, ecotopes

## Abstract

A comparative study of the kinetic characteristics (specific activity, initial and maximum rate, and affinity for substrates) of key enzymes of assimilatory sulfate reduction (APS reductase and dissimilatory sulfite reductase) in cell-free extracts of sulphate-reducing bacteria (SRB) from various biotopes was performed. The material for the study represented different strains of SRB from various ecotopes. Microbiological (isolation and cultivation), biochemical (free cell extract preparation) and chemical (enzyme activity determination) methods served in defining kinetic characteristics of SRB enzymes. The determined affinity data for substrates (i.e., sulfite) were 10 times higher for SRB strains isolated from environmental (soil) ecotopes than for strains from the human intestine. The maximum rate of APS reductase reached 0.282–0.862 µmol/min×mg^−1^ of protein that is only 10 to 28% higher than similar initial values. The maximum rate of sulfite reductase for corrosive relevant collection strains and SRB strains isolated from heating systems were increased by 3 to 10 times. A completely different picture was found for the intestinal SRB V_max_ in the strains *Desulfovibrio piger* Vib-7 (0.67 µmol/min × mg^−1^ protein) and *Desulfomicrobium orale* Rod-9 (0.45 µmol/min × mg^−1^ protein). The determinant in the cluster distribution of SRB strains is the activity of the terminal enzyme of dissimilatory sulfate reduction—sulfite reductase, but not APS reductase. The data obtained from the activity of sulfate reduction enzymes indicated the adaptive plasticity of SRB strains that is manifested in the change in enzymatic activity.

## 1. Introduction

Sulfate-reducing bacteria (SRB) are known to be the main producers of the biogenic hydrogen sulfide in the biosphere. They can be found in a number of natural ecotopes and in human-caused and industrial systems, such as sea sludge deposits, hydrothermal springs, freshwaters, soils, anaerobic muds, as well as wastewaters, oil and gas fields, on the surfaces of metal constructions of gas and oil communications, in oil tankers, thermal systems, sewers, reinforced concrete and metal installations, etc. [1,2,3,4,5,6,7,8]. SRB could live in animal and human intestines [9,10,11,12,13,14,15]. These bacteria were revealed as competitors with intestinal *Clostridia*, assimilating sulfates [16,17].

The fundamental metabolic feature of SRB is the hydrogen sulfide production as the result of dissimilatory sulfate reduction, i.e., sulfidogenesis [18,19,20]. Biogenic hydrogen sulfide produced by SRB in the industrial systems behaves as an aggressive corrosive agent, promoting the biodeterioration of steel [7]. In the human-caused systems, these bacteria shifted to a corrosive agent and are involved in the microbial corrosion processes [1]. Along with sulfate, sulfite can be a second alternative electron acceptor for SRB in the process of dissimilatory sulfate reduction. Under conditions of insufficient sulfate in the human or animal gut, sulfite is used by SRB and reduced to hydrogen sulfide. This ability of SRB can provide the overproduction of hydrogen sulfide and the emergence of aggressiveness of SRB in the intestine [4,5,6,21,22,23]. Overall, sulfate reduction is a primary driver of a carbon cycle and enzymes of sulfate reduction pathways catalyze to rate-limiting steps in the global sulfur cycle [24,25,26,27].

The sulfate-reduction pathway of SRB is catalyzed by three key enzymes: ATP sulfurylase, APS reductase, and sulfite reductase. The initial stage is the activation of sulfate by ATP sulfurylase enzyme (*sat*, EC 2.7.7.4), and the following reaction is the reduction of adenosine-5′-phosphosulfate (APS) to adenosine-monophosphate (AMP) and sulfite by adenylylsulfate reductase (*aps*, EC 1.8.4.9), and subsequent reduction to hydrogen sulfide by dissimilatory sulfite reductases (*dsr*, EC 1.8.99.3) [28]. Two electrons are necessary for the reduction of APS to AMP. This is a key step in the pathway of the sulfate assimilation and dissimilation, both in prokaryotic and eukaryotic cells. Sulfite reductase is capable of reducing sulfite to sulfide without the formation of intermediates. This reaction is also a terminal step in dissimilatory sulfate reduction. Among the enzymes of the dissimilatory sulfate reduction pathway, APS reductase and dissimilatory sulfite reductase are suitable indicators of this process in the environment [28,29]. Adenosine-5′-phosphosulfate reductase (EC 1.8.99.2; adenylyl-sulfate reductase or APS reductase) is one of the key enzymes of dissimilatory sulfate reduction in SRB.

DSR enzymes of various types occur in all organisms capable of reducing sulfite/sulfate during anaerobic respiration in all the SRB genera, especially of the *Desulfovibrio* genus [1,30,31,32], the sulfate-reducing archaea *Archaeoglobus fulgidus*, *Archaeoglobus profundus*, *Pyrobaculum islandicum* [33,34,35], while the dissimilatory type of sulfite reductase is inducible in the presence of sulfite in *Salmonella enterica* [36] and *Clostridium pasteurianum* [37], but the function of this enzyme is not clear. Thus, we can see that the process of sulfate reduction covers not only the process of hydrogen sulfide release, but also its assimilation, which can occur with the participation of the above enzymes [38].

In our previous study, we characterized the initial step of the dissimilatory sulfate-reduction pathway. The enzymatic parameters of ATP sulfurylase in cell-free extracts of SRB isolated from various ecotopes such as soils, corrosion products and human large intestine were determined in the research. The obtained results indicate significantly (*p* < 0.05) different rates of enzymatic reaction, catalyzed by ATP sulfurylase in the bacterial strains, isolated from various environmental ecotopes. Based on cluster analysis, the parameters of physiological and biochemical characteristics of SRB from different ecotopes were divided into three clusters that corresponded to the location of their isolation (soils, heating systems and human intestine). The sulfate reduction inhibition effects of the initial stage in SRB isolated from intestinal strains have also been shown. The process can be recognized as the unusual adaptation of SRB to the intestinal environment [39].

At present, the kinetic properties of enzymatic reactions in SRB with different physiological properties and isolated from various ecotopes have never been compared and described before. The aim of this study was to compare analysis of enzymatic activity of APS reductase and sulfite as the key enzymes in the final reaction of the sulfidogenesis of SRB, and to compare their kinetic parameters (initial and maximum rates of enzymatic reactions, and Michaelis constants) in cell-free extracts of SRB from various ecotopes. The results in the experiment conduction provide more information about the aggressiveness of SRB in different environments.

## 2. Materials and Methods

### 2.1. Bacterial Cultures and Cultivation

Sulfate-reducing bacteria (SRB) were isolated from various ecotopes, including soil, corrosion products and human feces. Isolation, purification and identification were previously described [40,41,42] and are presented in Table 1. Collection strains of *Desulfovibrio desulfuricans* DSM642 and *Desulfovibrio vulgaris* DSM644 (GenBank: AF418179.1) were isolated from corrosion products and soil near a gas-main and obtained from Deutsche Sammlung von Mikroorganismen und Zellkulturen GmbH (DSMZ) (Braunschweig Germany). *Desulfovibrio* sp. 10 (UCM B-11503) (GenBank: KC886400) were isolated from corrosion products of steel construction of DniproHES and were obtained from the Ukrainian Collection of Microorganisms at D.K. Zabolotny Institute of Microbiology and Virology of NAS of Ukraine (Kyiv, Ukraine), as well as corrosion strains: *Desulfovibrio* sp. TC2 (UCM B-11504), *Desulfotomaculum* sp. TC3 (UCM B-11505) and *Desulfomicrobium* sp. TC4 (UCM B-11506) were isolated from city heat system construction’s corrosion products (Kyiv, Ukraine). The intestinal SRB strains *Desulfovibrio piger* Vib-7 (GenBank: KT881309.1) and *Desulfomicrobium orale* Rod-9 (GenBank: MF939896.1) were isolated from the human large intestine (human feces) and obtained from at the Laboratory of Anaerobic Microorganisms of the Department of Experimental Biology at Masaryk University (Brno, Czech Republic).

Cultivation of SRB was performed in liquid modified Postgate C medium [40]. The highest sulfate concentration from 7.2 to 22.69 mmol L^−1^ was used in this modified Postgate C medium [31]. To adjust the pH (7.2–7.5), a sterile 10 mol L^−1^ solution of NaOH was used. The redox and anaerobic conditions were controlled by resazurin sodium (Oxoid, BR 0055B). Low redox potential (Eh = −100 ...−200 mV) for anaerobic condition was achieved by the addition of 2% ascorbic acid or 2% solution of sodium sulfide (1 mL L^−1^ of cultivation media). The tubes were filled with media, which were inoculated with SRB cultures (5% *v/v*), then closed by rubber plug to provide anaerobic conditions. The corrosive and soil SRB were cultivated at +28 °C for 7 days, while the intestinal bacteria were grown at +37 °C for 3 days.

### 2.2. Sulfate Determination

The content of sulfate in the medium was determined by the turbidimetric method right after the seeding and after 24 h of cultivation. Its essence lies in the precipitation of sulfate ions with the BaCl_2_ and turbidimetric determination of it in the form of BaSO_4_. For the suspension stabilization, glycerol was used. Suspension of 40 mg L^−1^ BaCl_2_ has been prepared in 0.12 mol L^−1^ HCl. The resulting solution was mixed with glycerol in a 1:1 ratio. To the 1 mL of sample supernatant after centrifugation at 5000× *g* at 23 °C (Hettich EBA 12 Centrifuge, Vlotho, Germany) was added 10 mL of prepared BaCl_2_:glycerol solution (in 1:1 ratio) and carefully stirred. The mixture was allowed to stand for 10 min and right after that the absorbance was measured at 520 nm (Spectrasonic Genesis 5, Berlin, Germany). As a control, the measurement was repeated with the same method using a cultivation medium. The calibration curve has been constructed with the same process. Calibration solutions have been prepared in distilled water at concentrations of 2, 4, 8, 16, 24, 32, 40, and 48 µmol L^−1^ of sodium sulfate [43].

### 2.3. Measurement of Hydrogen Sulfide

Production of hydrogen sulfide by SRB has been measured right after inoculating and after 24 h of cultivation spectrophotometrically. A 1 mL sample was added to 10 mL of a 5 g L^−1^ aqueous solution of zinc acetate. Right after this, 2 mL of a 0.75 g mL^−1^ of p-amino dimethylaniline in solution of sulfuric acid (2 mol L^−1^) was added. The mixture was left to stand for 5 min at room temperature. After that, 0.5 mL of 12 g L^−1^ solution of ferric chloride dissolved in 15 mmol L^−1^ sulfuric acid was added. After standing for another 5 min at room temperature, the mixture was centrifuged at 5000× *g* at 23 °C (Hettich EBA 12 Centrifuge, Vlotho, Germany). The absorbance of the centrifuged supernatant was determined to measure sulfide ions at a wavelength of 665 nm by spectrophotometer (Spectrasonic Genesis 5, Berlin, Germany). As a control, the measurement was repeated in the same method using a cultivation medium. The calibration curve was constructed with the same process. Calibration solutions were prepared in distilled water at concentrations of a 12.5, 25, 50, and 100 µmol L^−1^ of sodium sulfide [44].

### 2.4. Cell-Free Extracts

Cell-free extracts were prepared from the bacterial cell gained from the exponential phase of growth (1 day for intestinal strains, 7 days for corrosive-relevant strains). The bacteria were grown anaerobically in modified Postgate C liquid medium [31]. The cold extraction buffer (5 mol L^−1^ EDTA, 50 mmol L^−1^ potassium phosphate buffer, pH = 7.5) was added to centrifuged sediment cells to bind and depose heavy metal ions. After this procedure, the suspended bacterial cells containing 0.096–0.927 mg of protein × mL^−1^ were obtained. The cells were homogenized using the ultrasonic homogenizer (Bandelin SONOPULS GM 200, Berlin, Germany) at 20 kHz for 5 min at 0 °C. The soluble fractions were placed into centrifugal tubes and cell-free extracts were separated from the cell fragments by centrifugation for 30 min at 14,000× *g* and at 4 °C (Hettich EBA 12 Centrifuge, Vlotho, Germany). Supernatant was then used as cell-free extracts. The protein concentration in the cell-free extracts was determined by the Bradford method (1976) [45].

### 2.5. APS Reductase Activity

The activity of adenosine-5′-phosphosulfate reductase was measured by the AMP-dependent reduction of ferricyanide in the presence of sulfite. In a total of 3 mL of reaction mixture was contained Tris-HCl buffer (pH = 7.5; intestinal pH = 8.0), AMP (10 µmol), Na_3_Fe(CN)_6_ (4 µmol), Na_2_SO_3_ (10, 15, 20 mmol L^−1^ of SO_3_^2−^ in 5 mmol L^−1^ EDTA), and cell-free extract (50 µL). The incubation temperature was 27 °C (intestinal 35 °C) and the reaction was started by the addition of sulfite-EDTA solution. Ferricyanide reduction was spectrophotometrically (Spectrasonic Genesis 5, Berlin, Germany) measured at 420 nm. The enzyme can work in both directions [46].

### 2.6. Sulfite Reductase Activity

Sulfite reductase was assayed by the principles of Warburg’s effect based on the measurement of oxidation of NADH at 340 nm while SO_3_^2−^ was reduced [47]. The reaction mixture contained Tris-HCl buffer (pH = 7.5; intestinal pH = 8.0), Na_2_SO_3_ (5, 10, 15 mmol L^−1^ of SO_3_^2−^ in 5 mmol L^−1^ EDTA), NADH (0.2 mmol L^−1^), cell-free extract (50 µL) in total of 0.5 mL minus 27 µL of 1.2 mol L^−1^ HCl by which the reaction was started. The incubation temperature was 27 °C (intestinal 35 °C) and absorbance was read at 340 nm per minute for 10 min from the spectrophotometer (Spectrasonic Genesis 5, Berlin, Germany).

### 2.7. Kinetic Analysis

The study of kinetic properties of enzymes the APS reductase and sulfite reductase was performed as it described above. All experiments used to study the properties of these enzymes were performed using the initial rate V_0_ (linear accumulation of product (P) in time). The kinetic parameters that characterized APS reductase, and sulfite reductase such as Michaelis constant (K_m_^APS^, K_m_^Sulfite^) and maximum rate of these reactions (V_max_) were determined by the Lineweaver-Burk plots [48]. The obtained concentration dependence of the rate of enzymatic reaction on the studied reagent (SO_3_^2−^) was constructed in the coordinates (1/V on 1/S), where S is the concentration of the reagent (SO_3_^2−^), and V is the rate of enzymatic oxidation of SO_3_^2−^ at a concentration of SO_3_^2−^ for APS reductase, reduction of SO_3_^2−^ at a concentration of SO_3_^2−^ for sulfite reductase, respectively (Tables S1–S5).

### 2.8. Statistical Analysis

Statistical calculations of the results were carried out using the MS Office (2010), Origin 8.0 (https://www.originlab.com/) and Statistica 13 (http://www.statsoft.com/) software programs. Cluster analysis was performed by the single linkage method with the calculation of the Euclidean distances. Using the experimental data, the basic statistical parameters (mean: M, standard error: m, M ± m) were calculated. The research results were treated by methods of variation statistics using Student’s *t*-test. The significance of the calculated indicators of the line was tested by Fisher’s F-test. The approximation was accurate when *p* ≤ 0.05 [49]. The cluster analyses were conducted by the inclusion of the following parameters: enzymes’ specific activity, initial—maximum enzymatic reactions rates, and Michaelis constant. Statistical significance was also measured with the use of principal component analysis (PCA) that gave overall differences among compared groups. Statistical significance was also measured with the use of principal component analysis (PCA) by the inclusion of the same parameters as for the cluster analysis. PCA gave overall differences among compared groups.

## 3. Results

Two main metabolites of dissimilatory sulfate reduction processes were measured for studying SRB stains, isolated from various environments (Table 2).

**Figure 1:**
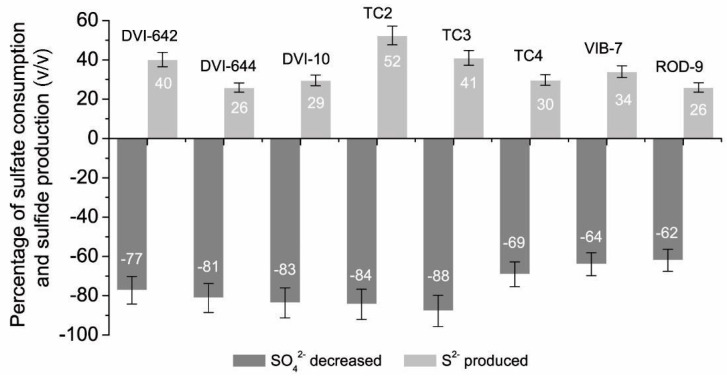
Changes of sulfate-ion accumulation and hydrogen sulfide production by sulfate-reducing bacteria (SRB) during 24 h of cultivation.

The initial concentration of sulfate ions in the nutrient medium was in the range of the following values: 3.53–7.27 mmol L^−1^. Initial concentrations of the sulfide-ion were from 0.20 to 1.01 mmol L^−1^. After 24 h of SRB cultivation, the final concentrations of sulfate and sulfide ions in the culture medium were in the range 0.83–1.58 mmol L^−1^ and 1.19–4.86 mmol L^−1^, respectively. The percentage ratio of consumed SO_4_^2−^ and produced S^2−^ are shown in Figure 1.

It was shown that in cultural media, the concentration of sulfate-ions decreased intensively by 62 to 88% (*v/v*), for all the SRB strains. Conversely, the production of sulfide ions for all SRB strains was increased by 26 to 52% (*v/v*). *Desulfotomaculum* sp. TC3 strain consumed the highest amount of SO_4_^2−^ (88% (*v/v*)) in comparison with other genera. Furthermore, the high consumption activity was shown by *Desulfovibrio* sp. TC2 (84% (*v/v*)), *Desulfovibrio* sp. 10 (84% (*v/v*)). The lowest consumption of sulfate ions was observed for the intestinal strains of *D. orale* Rod-9 and *D. piger* Vib-7; the reduction of sulfate ions in the culture medium was within the range: 62% (*v/v*) and 64% (*v/v*), respectively.

Among the tested strains, the maximum production of sulfide ions was revealed by bacteria isolated from the same ecotope—city heat system—*Desulfovibrio* sp. TC2 (52%), *Desulfotomaculum* sp. TC3. The lowest percentage (26% (*v/v*)) of sulfate was consumed by *D. vulgaris* DSM644. The increase in the production of sulfide ions (26 to 34%) by the intestinal strains of Rod-9 and Vib-7 was similar to that of *D. vulgaris* DSM644 and *Desulfovibrio* sp. 10 (26 to 30%).

It has to be emphasized that the production of sulfide ions did not correlate with the consumption of sulfate ions. The process of sulfate reduction may reflect the functional differences between SRB strains isolated from different habitats. Therefore, the kinetic parameters of key enzymes, including APS reductase and sulfite reductase, were subsequently analyzed.

**Figure 2:**
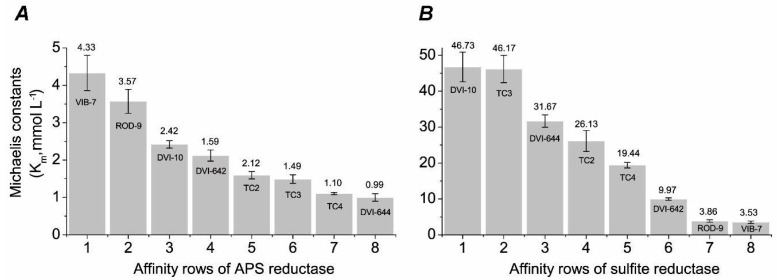
Comparison of the affinities of both enzymes of dissimilatory sulfate reductions in various SRB strains: APS reductase (**A**) and sulfite reductase (**B**).

Activated sulfate as APS is further reduced to sulfite by APS reductase and least to hydrogen sulfide by sulfite reductase [1,28]. These enzymes of dissimilatory sulfate reduction and their specific activities and kinetic parameters were calculated (Table 3, Figure 2).

The specific activity data for both enzymes were different for SRB strains from different ecotopes. Activity values for APS reductase and sulfite reductase were in the range of 0.113–0.340 and 0.028–0.516 U/mL (0.8333–5.666 and 0.466–8.600 nkat), respectively. The highest activity of APS reductase was detected in cell-free extracts of the following strains: *D. piger* Vib-7 (5.666 nkat), *D. vulgaris* DSM644 (3.900 nkat) and *Desulfotomaculum* sp. TC3 (3500 nkat) isolated from different ecotopes. However, according to the activity of the final step of the dissimilatory sulfate reduction enzyme (sulfite reductase), the highest activity is shown in the cell-free extracts of *Desulfovibrio* sp. 10 (8600 nkat), *D. vulgaris* DSM644 (7050 nkat) collection strains, and *Desulfotomaculum* sp. TC3 (8383 nkat) and *Desulfovibrio* sp. TC2 (6000 nkat) isolated from city heat networks. The lowest activity of sulfite reductase was found in intestinal strains of SRB, 0.466 and 0.533 nkat, which is 16.1 and 18.4 times lower, respectively, according to the value of the activity of *Desulfovibrio* sp. 10.

The affinity rows were shown to estimate the substrate specificity, evidenced by the value of the Michaelis constant (Figure 2). It is visible how much the Michaelis constant differs in every SRB strain: it varied between 0.99 and 4.33 mmol L^−1^ for APS reductase and 3.53 and 46.73 mmol L^−1^ for sulfite reductase. This parameter differs in representatives of even one genus, *Desulfovibrio*. By affinity for the substrate for the enzyme APS reductase, the highest affinity was shown in the intestinal strains such as *D. piger* Vib-7 (4.33 mmol L^−1^), *D. orale* Rod-9 (3.57 mmol L^−1^), and SRB *Desulfovibrio* sp. 10 (2.42 mmol L^−1^). For the intestinal *D. piger* Vib-7 strain, it is shown that the high specific activity of APS reductase (5.666 ± 0.483 nkat) coincides with the affinity for the substrate, expressed by the Michaelis constant. Although *D. vulgaris* DSM644 had the higher specific activity of APS reductase, the value of K_m_^APS^ (0.99 mmol L^−1^) was the lowest K_m_^APS^ (0.99 mM) among the studied strains. Rod-9 has the lowest specific activity of APS reductase, but a significant affinity to APS substrate (K_m_^APS^ = 3.57 mmol L^−1^). The specific activity of APS reductase for SRB strains isolated from different ecotopes did not match the affinity for the substrate.

The data of activity of the enzyme in the final step of the sulfate reduction process corresponded to the calculated Michaelis constant values, and the affinity for the substrate (sulfite) was the highest in *Desulfovibrio* sp. 10 (46.73 mmol L^−1^), *Desulfotomaculum* sp. TC3 (46.17 mmol L^−1^) and *D. vulgaris* DSM644 (31.67 mmol L^−1^). It is characteristic that the smallest specific activity of sulfite reductase was detected in intestinal strains of SRB (0.466–0.533 nkat) and the least affinity for the substrate was determined according to the data of the Michaelis constant (3.53–3.86 mmol L^−1^). Therefore, the specific sulfite reductase activity for all tested SRB strains isolated from different ecotopes practically coincided with the affinity for the substrate.

**Figure 3:**
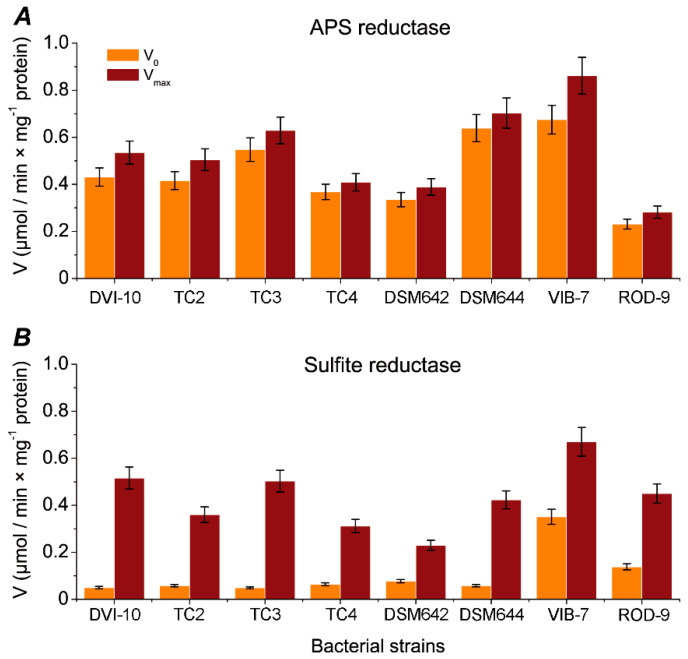
Comparison of sulfate reduction process reactions in various SRB strains: APS reductase (**A**) and sulfite reductase (**B**).

Important indicators of the reaction kinetics involved in the processes of dissimilatory sulfate reduction are the rates of product conversion reactions. The initial (V_0_) and maximum (limiting) V_max_ rates of enzymatic reactions were determined (Figure 3). The SRB strains have an initial rate of V_0_ of substrate consumption for APS reductase of 0.231–0.675 µmol/min×mg^−1^ protein. The highest initial velocity values of this enzyme were found in *D. vulgaris* strain DSM644 (0.639 µmol/min × mg^−1^ protein) and *D. piger* Vib-7 (0.675 µmol/min × mg^−1^ protein). The maximum rate of APS reductase reached 0.282–0.862 µmol/min × mg^−1^ of protein, which is only 10 to 28% higher than similar initial values.

For sulfite reductase, V_0_ was 0.049–0.076 µmol/min × mg^−1^ protein for corrosion-active strains isolated from collection and the strains isolated from heating systems, and the initial intestinal SRB rate was quite different (0.138 and 0.351 µmol/min × mg^−1^ protein for *D. piger* Vib-7 and *D.orale* Rod-9, respectively). It is significantly higher by 2.81–4.61 times than the minimum initial velocity of 0.049 µmol/min×mg^−1^ protein for DVI-10 strain. The maximum rate of sulfite reductase reached values of 0.23–0.516 µmol/min×mg^−1^ protein (for corrosion-active collection strains and SRB strains isolated from heating systems). The rate increased by 2.98–10.32 times. However, a completely different picture was found for intestinal SRB, where the V_max_ in strains for *D. piger* Vib-7 (0.067 µmol/min × mg^−1^ protein) and *D. orale* Rod-9 (0.045 µmol/min×mg^−1^ protein) was decreased significantly by 5.24 and 3.06 times, respectively.

**Figure 4:**
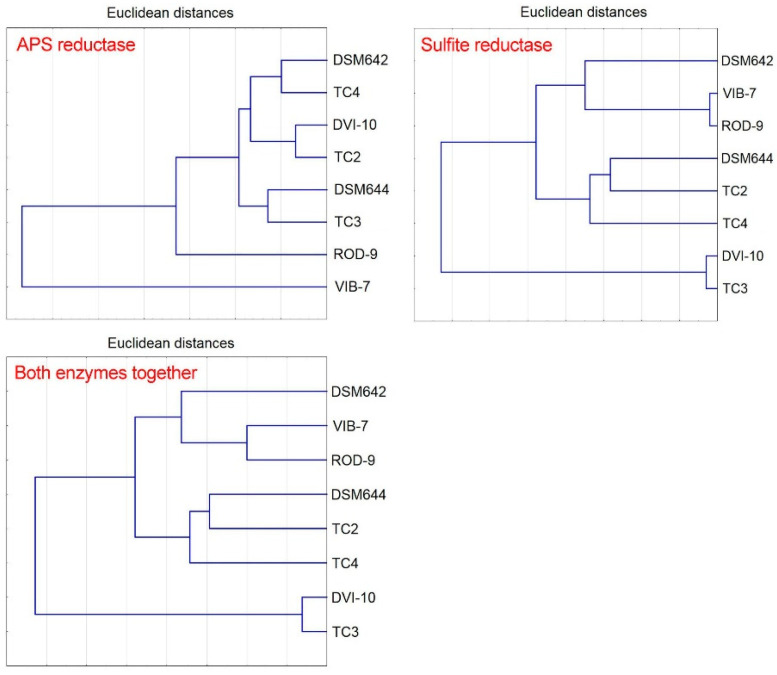
Cluster analysis of the kinetic parameters of sulfate reduction process.

To analyze the affinity of SRB strains according to the kinetic parameters of two enzymes of dissimilatory sulfate reduction, a cluster analysis was performed. Figure 4 shows the cluster analysis results. The results indicate differences among evaluated bacterial strains toward applied enzymes (APS reductase, sulfite reductase, and both enzymes together). The APS reductase parameter divided the studied strains into twoclusters. The first cluster was divided into three sub-clusters: the first TC4 and DSM642; the second DVI-10 and TC2; the third DSM644 and TC3. The intestinal strain ROD-9 joined a cluster, but divides from sub-clusters. In addition, SRB Vib-7 was separated and did not belong to any cluster. Sulfite reductase parameters formed following separated clusters: the first cluster contained VIB-7 and ROD-9 with additional strain DSM642; the second cluster contained DSM644, TC2 and TC4; and the third cluster contained DVI-10 and TC3. Almost the same results (as sulfite reductase) were obtained when both parameters were included in one cluster analysis (Figure 4). Therefore, these results indicate that the determinant in the cluster distribution of strains of SRB is the activity of the terminal enzyme of dissimilatory sulfate reduction—sulfite reductase, not APS reductase. This difference can be caused by the presence of SRBs in certain environments (soils, corrosion products or human intestine), which are selective for intestinal SRB strains.

**Figure 5:**
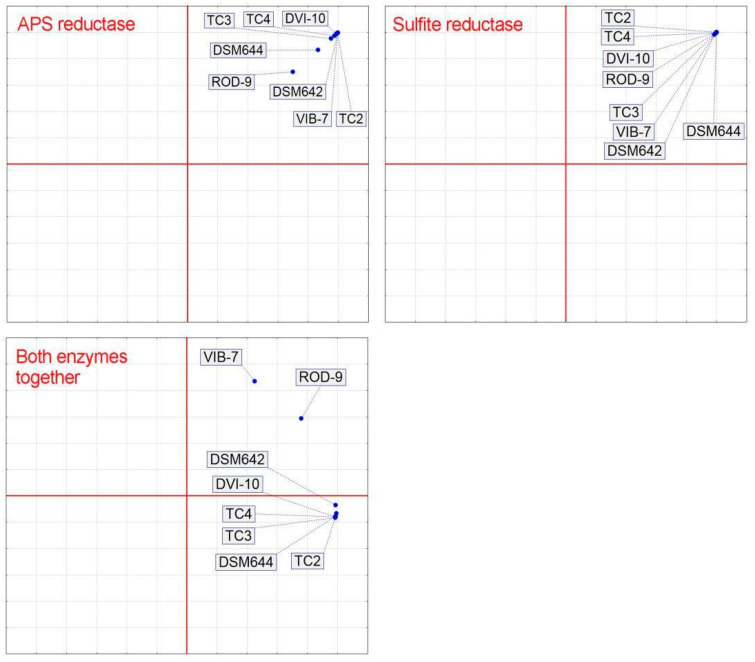
Principal component analysis of the kinetic parameters of sulfate reduction process.

Principal component analysis (PCA) was used to separate SRB strains (TC3, TC4, DVI-10, DSM642, VIB-7, TC2, DSM644 and ROD-9) according to their parameters of APS and sulfite reductases separately and together (Figure 5).

The following strains, TC3, TC4, DVI-10, DSM642, VIB-7 and TC2, formed one cluster by the parameters of APS reductase. DSM644 and ROD-9 were separated from this cluster. Conversely, no differences were observed between SRB strains in sulfite reductase parameters. Based on the activity of both enzymes (APS and sulfite reductase), VIB-7 and ROD-9 were separated from the rest of the SRB.

## 4. Discussion

The sulfate reduction enzymes are located in the cytoplasm and peripheral plasma. The sulfate ions can be transported into the cells simultaneously with protons and some sulfate-reducing bacteria, absorbing sulfate from the flow of sodium ions [1]. This high activity in the cell-free extracts may be caused by determining it in a crude sample of intestinal SRB grown only in a selective medium for SRB. On the other hand, it can also be based on their inhabitant location, which is the large intestine of rats and mice [22,23].

The second key enzyme, APS reductase, is mainly found in the dissimilatory SRB, such as species of *Desulfovibrio* and *Desulfotomaculum* genera, and in some oxidizing sulfite to sulfate *Thiobacillus* spp. [50]. The specific enzymatic activity (0.095 U × mg of protein^−1^) of this enzyme was assayed by Huisingh et al. (1974) [46] for a *Desulfovibrio* sp. strain isolated from sheep rumen and described. Similar activity was also observed in a crude cell-free extract of *D. desulfuricans* (0.08 U × mg of protein^−1^) [50]. Our obtained data about specific activity values for APS reductase were in the range 0.113–0.340 U/mL (0.8333–5.666 nkat). It can be seen that the specific activity of APS reductase in cell-free extracts of the strains was two orders of magnitude higher than in the literature. Enzymatic mechanism of sulfite reduction in SRB isolated from the environment was elucidated by Isimoto and Yagi (1960) and Seki et al. (1978) [51,52]. The specific activity of sulfite reductase from *D. vulgaris* was determined to be 0.19 U × mg of protein^−1^ in both fractions after DEAE-Sephadex column chromatography [53]. On the other hand, higher activity (0.99 U × mg of protein^−1^) of this enzyme was detected in supernatants of crude extracts from *Desulfovibrio* spp. [50]. The specific enzymatic activities obtained from our studied SRB samples ranging from 0.028 ± 0.001 to 0.516 ± 0.04 U × mg of protein^−1^ were in the range of activities mentioned above. Intestinal SRB isolated from the large intestine of rodents (rats and mice) show differences in enzymatic activity for sulfite reductase in each sample taken (0.317 ± 0.037 to 0.702 ± 0.023 U × mg of protein^−1^), even in the similar group of SRB strains. The maximum rate of sulfite reductase in intestinal strains (Vib-7 and Rod-9) was lower than the initial rate in the corrosion-active SRB strains, in which the maximum rate was expected to be higher than the initial one (see Figure 3).

Generally, a reduction of sulfate became a dominant biological process in the water environment (oceans and deposits), resulting in sulfidic anoxic conditions from 2.5 to 0.6 billion years ago [54,55]. Currently, it is well known that SRB are able to colonize habitats that are different from the aquatic anaerobic environment where microorganisms have existed for a long time; in particular, the intestinal tract of animals (sea urchins, rodents, cows) and humans [56,57], where microorganisms need to adapt to new living conditions. The confirmation of this is that affinity data for substrates indicate that high affinity was shown for sulfite by SRB strains isolated from natural ecotopes, mainly in the soil environment. Additionally, the fact that the content of sulfates/sulfites in the aquatic environment (in particular in marine) (up to 2.7 g L^−1^ or soluble sulfate-ion 0.0283 moles kg^−1^ water) [3,58] differs from those present in the intestinal lumen and that come from food and beverages [59] may be a selective factor leading to changes in the functioning of the enzymatic system. Subseafloor microbial ecosystems transform sulfur compounds; approximately 11.3 moles of sulfate are microbially reduced each year, accounting for the oxidation of 12 to 29% of the organic carbon flux to the seafloor [3]. Dissimilatory sulfate reduction by marine sediments largely depends on the availability of sulfate supplied from seawater [60,61].

The sulfite reductases belong to a family of proteins that also include the assimilatory sulfite and nitrite reductases [54,62]. These enzymes reduce sulfite to a mixture of tritionate, thiosulfate and sulfide in proportions that depend on the reaction (environmental) conditions [63]. In some *Desulfovibrio* spp. strains, through the regulation of nitrate and nitrite oxidation, induction by nitrate and repression by sulfate is possible (i.e., sulfate reduction in the presence of nitrates is suppressed) [64]. It is known that the same reactions in SRB can work in energetic and constructive directions, that is, be reversible [54]. Extensive metabolic plasticity of SRB is making them capable to use other reduced sources of sulfur in the absence of sulfate. In addition, they are capable of performing nitrate reduction, nitrite reduction, acetogenic reactions and the transfer of molecular hydrogen to other microorganisms. All these qualities bring the SRB closer to other anaerobes and provide space for constructing evolutionary circuits and information transmission pathways between different anaerobic groups [28,65,66,67]. As discussed in the studies by Klein (2001), similar patterns of insertions and deletions in ApsA sequences of donor and recipient lineages provide additional evidence for lateral gene transfer. From a subset of reference strains (*n* = 25), a fragment of the dissimilatory sulfite reductase genes (*dsrAB*) that have recently been proposed to have undergone multiple lateral gene transfers [68] was also amplified and sequenced. Phylogenetic comparison of DsrAB- and ApsA-based trees suggests a frequent involvement of Gram-positive and thermophilic SRB in lateral gene transfer events among the SRB. Further patterns of lateral gene transfer (LGTs) in SRB exist; these may also be linked to their ecophysiology. Most recipients of xenologous *dsrAB* and *apsA* genes are thermophilic [28]. We can assume that the likely intestinal SRB strains have acquired some adaptation to the conditions of the intestinal tract that is significantly different both in the content of sulfate and in elevated temperature from the conditions of the soil and water environments.

In view of the fact that *Salmonella enterica* [36] and *Clostridium pasteurianum* [37] utilize sulfate in the assimilatory sulfate reduction process and the main product is not hydrogen sulfide released from the outside, but rather is a cysteine, which is a part of sulfur-containing proteins. A dissimilatory sulfite reductase was purified from *Bilophila wadsworthia* RZATAU and is involved in energy conservation by reducing sulfite to sulfide, during the degradation of taurine as an electron acceptor [69]. This enzyme is also found in some phototrophic and chemotrophic sulfur oxidizers, where it is proposed to operate in the reverse direction (reverse sulfite reductase, rDSR) [54,70]. To confirm this, a recombinant Vib-7M strain was constructed that contained gene *CysK* coded O-acetyl-serine(thiol)lyase, which is an enzyme catalyzing the reaction of inorganic sulfide with O-acetyl-serine to form the S-containing amino acid l-cysteine [56]. Mutant strains are capable of higher speeds (1.5 times) using sulfate compared with the strains that are in the intestines of animals with colitis. Therefore, it can be assumed that, unlike strains isolated from the soil, including strains DVI-10, DSM642 and DSM644, for the intestinal strains Vib-7 and ROD-9, there is competition in trying to restructure the metabolism similar to that prevalent in the microorganisms of the intestinal microbiota. This is manifested in a decreased rate of sulfite reductase, and a decreased affinity to the substrate (sulfites). For intestinal strains of SRB, similar results were obtained for the first step of sulfate-reduction pathway, i.e., ATP sulfurylase. We discussed that this decrease in the maximum rate of the enzyme could show the inhibition of the enzymatic process of sulfate accumulation in the bacterial cells at the end of the reaction [39]. Therefore, it is possible that, in addition to competition for substrates with methanogens by the intestinal SRB strains, inhibition of the final stages of sulfate reduction may occur [71,72].

The cluster analysis data confirm changes in the adaptation of SRB strains to different existence conditions, and indicate that the terminal enzyme (sulfite reductase) is crucial in the adaptation of bacterial cells to environmental conditions, namely changes in their specific activity.

## 5. Conclusions

A comparative study of the kinetic characteristics (specific activity, maximum rate, and affinity of the substrates) of dissimilatory sulfate reduction key enzymes (in particular APS reductase and dissimilatory sulfite reductase) was carried out. Determined affinity data for substrates indicate a high affinity for sulfite by SRB strains isolated from natural ecotopes, mainly in the soil environment. The maximum rate of sulfite reductase in intestinal strains (Vib-7 and Rod-9) is lower than the initial rate, and also than the initial one in the corrosive relevant SRB strains. The determinant in the cluster distribution of SRB strains is the activity of the terminal enzyme of dissimilatory sulfate reduction—sulfite reductase, not APS reductase. This difference could be caused by the presence of sulfite-reducing bacteria in environments (soils, corrosion products or human intestine) in which one is selective for intestinal SRB but, without isolation, the contamination of sulfite-reducing bacteria can be expected.

## Figures and Tables

**Table 1:** Characteristic of the sulphate-reducing bacteria (SRB) from various ecotopes.

No	Bacterial Strains	Source of Isolation (Country of Origin)	References
1	*Desulfovibrio desulfuricans* DSM642	Corrosion product (DSMZ collection, Germany)	[40]
2	*Desulfovibrio vulgaris* DSM644	Soil (DSMZ collection, Germany)	
3	*Desulfovibrio* sp. 10 (UCM B-11503)	Corrosion products of steel construction of DniproHES (Ukraine)	[41]
4	*Desulfovibrio* sp. TC2	Corrosion products and slime from heat system construction (Kyiv, Ukraine)	[42]
5	*Desulfotomaculum* sp. TC3		
6	*Desulfomicrobium* sp. TC4		
7	*Desulfovibrio piger* Vib-7	Human feces from people with colitis (Brno, Czech Republic)	[11]
8	*Desulfomicrobium orale* Rod-9		

**Table 2:** Hydrogen sulfide production during accumulation of sulfates by SRB cultures from various ecotopes (M ± m, *n* = 3; where “M” is average, “m” is mean, and “*n*” is the number of repetitions).

Bacterial Cultures	SO_4_^2−^ at the Beginning (mmol L^−1^)	SO_4_^2−^ after 24 h Cultivation (mmol L^−1^)	S^2−^ at the Beginning (mmol L^−1^)	S^2−^ after 24 h Cultivation (mmol L^−1^)
*D. desulfuricans* DSM642	6.28 ± 0.446	1.43 ± 0.092	0.72 ± 0.066	4.53 ± 0.080
*D. vulgaris* DSM644	7.27 ± 0.379	1.37 ± 0.101	0.43 ± 0.040	4.18 ± 0.248
*Desulfovibrio* sp. 10	5.05 ± 0.121	0.83 ± 0.055	0.44 ± 0.038	3.83 ± 0.265
*Desulfovibrio* sp. TC2	5.56 ± 0.192	0.87 ± 0.193	1.01 ± 0.092	4.86 ± 0.289
*Desulfotomaculun* sp. TC3	6.35 ± 0.142	0.78 ± 0.028	0.78 ± 0.071	4.76 ± 0.298
*Desulfomicrobium* sp. TC4	5.43 ± 0.292	1.58 ± 0.156	0.43 ± 0.039	3.61 ± 0.256
*Desulfovibrio piger* Vib-7	3.50 ± 0.032	1.25 ± 0.110	0.30 ± 0.027	2.21 ± 0.192
*Desulfomicrobium orale* Rod-9	3.53 ± 0.030	1.34 ± 0.123	0.20 ± 0.018	1.97 ± 0.185

**Table 3:** Kinetics parameters of key enzymes of the dissimilatory sulfate-reduction pathway (M ± m, *n* = 3).

Sample	Specific Activity (nkat)	
	APS Reductase	Sulfite Reductase
DSM642	2.166 ± 0.020	3.833 ± 0.35
DSM644	3.900 ± 0.034	7.050 ± 0.65
DVI-10	2.967 ± 0.016	8.600 ± 0.40
DVI-TC2	2.800 ± 0.022	6.000 ± 0.23
DTM-TC3	3.500 ± 0.034	8.383 ± 0.37
DMI-TC4	2.226 ± 0.019	5.200 ± 0.48
VIB-7	5.666 ± 0.483	0.533 ± 0.026
ROD-9	1.833 ±0.200	0.466 ± 0.022

## Supplementary Materials

The following are available online at https://www.mdpi.com/2218-273X/10/6/921/s1. Table S1: Oxidation of photochemically reduced methyl viologen; Table S2: Hydrogen uptake (µmoles), Table S3: Oxidation of NADH (mM/mg of protein) in time (min) via sulfite reductase; Table S4: The initial (V_0_) and maximum (limiting) V_max_ rates of enzymatic reactions; Table S5: Affinity rows of APS reductase and sulfite reductase in various strains of sulfate reducing bacteria.
