# Supplementary material for: Adenosine-5′-Phosphosulfate- and Sulfite Reductases Activities of Sulfate-Reducing Bacteria from Various Environments
Source: Biomolecules. 2020 Jun 17;10(6):921. doi: 10.3390/biom10060921 (PMC7357011; doi:10.3390/biom10060921)
Supplement: Supplementary file 1 [file biomolecules-10-00921-s001.pdf]

SUPPLEMENTARY MATERIALS

Table 1S. Oxidation of photochemically reduced methyl viologen

Time (min)	Ferricyanide reduction (µmol/mg protein)	
	VIB-7	ROD-9
5	2.9	1
10	5.3	1.7
15	7.149	2.3
20	8.8	2.970
25	10.1	3.207
30	10.2	3.3

Table 2S. Hydrogen uptake (µmoles)

Time (min)	VIB-7	ROD-9
5	0.9	0.49
10	1.37	0.678
15	1.51	0.939
20	1.59	1.04
25	1.69	1.165
40	1.77	1.24
60	1.84	1.34
80	1.86	1.36
100	1.88	1.36

Table 3S. Oxidation of NADH (mM/mg of protein) in time (min) via sulfite reductase

Time (min)	DVI-10	TC2	TC4	DVI-642	DVI-644
1	0.930	0.763	1.178	1.102	1.044
2	0.501	0.510	0.691	0.572	0.576
3	0.333	0.315	0.455	0.369	0.367
4	0.240	0.231	0.318	0.248	0.249
5	0.179	0.171	0.232	0.207	0.181
6	0.135	0.131	0.182	0.168	0.133
7	0.101	0.115	0.140	0.144	0.108
8	0.090	0.093	0.115	0.115	0.089
9	0.074	0.089	0.092	0.104	0.087
10	0.059	0.079	0.080	0.091	0.075

Table 4S. The initial (V_0) and maximum (limiting) V_{max} rates of enzymatic reactions

Strains	APS reductase		Sulfite reductase	
	V_0	V_{max}	V_0	V_{max}
DVI-10	0.431	0.535	0.05	0.516
TC2	0.416	0.505	0.058	0.36
TC3	0.548	0.629	0.049	0.503
TC4	0.368	0.409	0.064	0.312
DSM642	0.335	0.389	0.077	0,23
DSM644	0.639	0.703	0.058	0.423
VIB-7	0.675	0.862	0.351	0.67
ROD-9	0.231	0.282	0.138	0.45

Table 5S. Affinity rows of APS reductase and sulfite reductase in various strains of sulfate reducing bacteria

Michaelis constants (K_m, mM)			
**Strains**	**APS reductase**	**Strains**	**Sulfite reductase**
**VIB-7**	4.33	**DVI-10**	46.729
**ROD-9**	3.57	**TC3**	46.171
**DVI-10**	2.419	**DSM644**	31.669
**DSM642**	2.121	**TC2**	26.126
**TC2**	1.594	**TC4**	19.4
**TC3**	1.49	**DSM642**	9.97
**TC4**	1.103	**ROD-9**	3.86
**DSM644**	0.999	**VIB-7**	3.53
